# Impact of Varying Soybean Oil Concentrations on the Cytokine Profile of Skeletal Muscle and Liver of Pigs: A Systems Biology Approach

**DOI:** 10.1002/age.70129

**Published:** 2026-06-21

**Authors:** Fernanda Nery Ciconello, Julia Dezen Gomes, Simara Larissa Fanalli, Bárbara Silva‐Vignato, Andrezza Maria Felício‐Ament, Vivian Vezzoni de Almeida, Cristina Tchorny Moncau‐Gadbem, James Eugene Koltes, Christopher Keith Tuggle, Aline Silva Mello Cesar

**Affiliations:** ^1^ “Luiz de Queiroz” College of Agriculture University of São Paulo Piracicaba Brazil; ^2^ School of Animal Science and Food Engineering University of São Paulo Pirassununga Brazil; ^3^ School of Veterinary and Animal Science Goiás State University Goiânia Brazil; ^4^ School of Veterinary Medicine and Animal Science University of São Paulo Pirassununga Brazil; ^5^ Department of Animal Science Iowa State University Ames Iowa USA

**Keywords:** ELISA, inflammation, lipids, swine, WGCNA

## Abstract

Lipids are important for metabolic processes, mainly cytokine regulation in the skeletal muscle and liver. Considering the soybean oil concentration, a weighted gene co‐expression network analysis (WGCNA) was used to perform an integrative analysis in skeletal muscle and liver tissues. Using this approach, this research aimed to investigate the integrative response to soybean oil supplementation in two different tissues to discover molecular responses, especially inflammatory responses. For this integrative analysis, sample collection, RNA sequencing, and WGCNA were carried out on immunocastrated male *Large White* pigs supplemented with 1.5% or 3.0% soybean oil. Co‐expression of some genes was revealed, such as those related to lipid metabolism and phosphotransferase activities in the skeletal muscle, or oxidative stress and methylation in the liver. Thus, we demonstrated that soybean oil supplementation leads to responses in skeletal muscle and liver, revealing that this supplementation improves the metabolism and inflammatory responses of healthy pigs.

## 1 Introduction

The addition of fats to the diet plays a key role for pigs in the growing‐finishing phases, due to the increased energetic content, enhancing the animals' development (Okrouhlá et al. 2018; Wealleans et al. 2021). Using vegetable oils in the food of pigs has been intensified in the last decades, promoting more efficient growth and improvement of the pork's nutritional quality (Almeida, Silva, Schinckel, et al. 2021; Liu et al. 2018; Wealleans et al. 2021). Among various sources of vegetable oils, soybean oil is one of the most used because of its wide availability, nutritional profile, and low cost (Alencar et al. 2021; Okrouhlá et al. 2018). Besides being rich in essential fatty acids, such as linoleic and linolenic acids, soybean oil has important functions in the metabolic processes of pigs, mainly in the lipid and energy metabolisms (Almeida, Silva, Schinckel, et al. 2021; Gaffield et al. 2022). Moreover, these essential fatty acids are directly associated with cytokine regulation in the hepatic and skeletal muscle tissues (Ciconello et al. 2025, 2026; Fritsche 2015), and it is crucial to study these interactions to understand and improve their effects on the health and performance of animals.

Many studies have investigated the impact of fatty acids in soybean oil on metabolism and inflammatory health (Liu 2015; Okrouhlá et al. 2018). The relationship between polyunsaturated fatty acids and their inflammatory response through cytokine regulatory mechanisms has been elucidated (Duan et al. 2014; Yao et al. 2012). It is well‐known that fatty acids are important in the inflammatory response. In spite of that, few studies have analyzed the effect of different concentrations of soybean oil on the cytokine profile in various tissues. In addition, the scientific community lacks an integrative analysis that broadly explores the interaction between skeletal muscle and liver, considering the inflammatory response and lipid metabolism.

To fill this gap, the Weighted Gene Co‐expression Network Analysis (WGCNA) appears as a powerful tool to study complex interactions in the different tissues and to identify co‐expression patterns associated with traits related to health (Kadarmideen 2016; Zhao et al. 2022). This approach allows the building of networks that make a connection between co‐expressed genes and traits of interest, enabling an integrative understanding of how oil concentrations can affect the body's system (Fanalli et al. 2024; Zhao et al. 2024). Moreover, the WGCNA application can identify clusters of genes related to biological, cellular, or molecular processes of different tissues and the regulation of cytokines, lipid metabolism, and inflammation (Ciconello et al. 2025, 2026; Fanalli et al. 2024; Zhao et al. 2020).

This study aimed to evaluate the impact of different concentrations of soybean oil in male pigs, using RNA‐Sequencing (RNA‐Seq) data from skeletal muscle (*Longissimus lumborum*) and hepatic tissues using the WGCNA to investigate the molecular integrative responses. The obtained results have crucial implications for better understanding the effects of varying concentrations of an important ingredient on the pigs' diets in metabolic health, especially on the inflammatory response and on the development of diseases related to skeletal muscle and liver.

## 2 Materials and Methods

### 2.1 Livestock Farming and Sample Collection

A total of 18 immunocastrated male pigs ( *Sus scrofa* , purebred Large White) were randomly allocated to two dietary treatments (*N* = 9 per group). The animals were fed a corn–soybean meal diet supplemented with either 1.5% soybean oil (SOY1.5—control diet) or 3.0% soybean oil (SOY3.0—experimental diet) (Almeida, Silva, Schinckel, et al. 2021). Feed nutritional formulation was based on Rostagno et al. (2011). The water and food were kept *ad libitum* for each pen containing the animals during the trial. The statistical power was calculated based on Duan and coworkers (Duan et al. 2014). The experimental animals were the offspring of 32 sows and three boars. The beginning of the trial occurred when the pigs were 71 days of age during the growing‐finishing phases. All pigs were genotyped for the halothane mutation (*RYR1* gene), and this validation showed that all the animals were homozygous negative (NN) to avoid confounding effects on meat quality, since the presence of this mutation is known to predispose pigs to Porcine Stress Syndrome (PSS).

Diets were formulated to provide similar levels of digestible and metabolizable energy, without the inclusion of additives. The piglets were immunocastrated with two doses of Vivax (Pfizer Animal Health, Parkville, Australia), following the manufacturer's recommendations. After 98 days of trial, at 169 ± 7 days of age, the pigs were slaughtered with an average weight of 132.06 kg for SOY1.5 and 132.73 kg for SOY3.0, with a standard deviation of 10.31, with no statistical difference.

At slaughter, samples of skeletal muscle *L. lumborum* and the right lobe of the liver were collected during the evisceration step. These samples were quickly snap‐frozen in liquid nitrogen to be stored in an ultra‐freezer (−80°C) for subsequent RNA extraction, RNA‐Sequencing (RNA‐Seq), and WGCNA. The cytokines interleukin (IL)‐10, interferon (IFN)‐γ, IL‐1β, IL‐6, IL‐18, and tumor necrosis factor (TNF)‐α were measured in both tissues, muscle and liver, by Milliplex Map Kit (Merck KGaA, Darmstadt, Germany), a multiplex sandwich enzyme linked‐immunosorbent assay (ELISA), according to the manufacturer's instructions. All six cytokines above were chosen because of the results obtained from differentially expressed genes in a previous study of our group (Fanalli et al. 2023; Fanalli, da Silva, Gomes, Ciconello, et al. 2022) and another group (Chen et al. 2019). These procedures follow the ARRIVE Guidelines 2.0 (https://arriveguidelines.org).

### 2.2 Total RNA Extraction and RNA‐Seq

Total RNA was extracted from skeletal muscle and liver using the RNeasy Mini Kit (QIAGEN, Hilden, Germany) based on the manufacturer's recommendations. The quantification, integrity, and purity were assessed by the Nanodrop 1000 (Thermo Fisher Scientific, Wilmington, DE, USA) and Bioanalyzer 2100 (Agilent, Santa Clara, CA, USA) systems. RNA integrity number (RIN) > 7.0 was measured for both tissues.

For RNA‐seq library preparation, 2 μg of total RNA was processed following the TruSeq RNA Sample Preparation Kit v2 protocol (Illumina, San Diego, CA, USA). The average library size was determined using the Agilent 2100 Bioanalyzer (Agilent, Santa Clara, CA, USA). Library quantification was performed by qPCR with the KAPA Library Quantification Kit (KAPA Biosystems, Foster City, CA, USA). Quantified libraries were diluted, barcoded, and pooled, then distributed across five lanes of the flow cell using the TruSeq DNA CD Index Plate and the TruSeq PE Cluster Kit v4‐cBot‐HS (Illumina, San Diego, CA, USA). A TruSeq Stranded mRNA Sample Preparation Kit, with PolyA selection for ribo depletion protocol, was applied. Sequencing was conducted on the Illumina HiSeq 2500 platform in paired‐end mode (2 × 100 bp) with the TruSeq SBS Kit v4‐HS (200 cycles), according to the manufacturer's instructions. Each sample yielded more than 30 000 reads (Fanalli, da Silva, Gomes, de Almeida, et al. 2022).

### 2.3 Quality Control, Alignment, and Normalization

FastQC version 0.11.8 [http://www.bioinformatics.bbrc.ac.uk/projects/fastqc/] was used to analyze and check the quality of raw reads. The next step was to remove sequences of barcodes, indexes, adaptors, and low‐complexity reads by using TrimGallore version 0.6.5 [http://www.bioinformatics.babraham.ac.uk/projects/trim_galore]. Reads used for the alignment procedure were filtered to have a Phred score > 33 and a length of > 70 nucleotides. After selecting high‐quality reads, they were aligned to the pig reference transcriptome (derived from the *Sus scrofa* 11.1 genome assembly and annotation available at Ensembl [http://www.ensembl.org/Sus_scrofa/Info/Index]) using Bowtie2 version 2.4.3 along with the RNA‐Seq by Expectation–Maximization (RSEM) version 1.3.3 pipeline for the estimation of expression values (Li and Dewey 2011). Prior to WGCNA, we applied a filter to each sample to remove the genes with no expression level in 80% of all samples.

### 2.4 Adjustment of Phenotypes

The phenotypes considered were six cytokines: IL‐10, IFN‐γ, IL‐1β, IL‐6, IL‐18, and TNF‐α. The effect of sire was fixed according to the previous analysis, fully described by Ciconello et al. (2025). The model is identified by Equation (1), which was fitted separately for skeletal muscle and liver.

(1) $${\hat{Y}}^{*}=\mu +y-X\hat{\beta }+\varepsilon$$

${\hat{Y}}^{*}$ is the adjusted/corrected phenotype; *μ* the overall mean phenotype; *y* is the vector of phenotypes; *X* is the incidence matrix for fixed effects; $\hat{\beta }$ is the fixed effect vector, which considered only sire; *ε* is the residual vector.

### 2.5 WGCNA

Weighted Gene Co‐expression Network Analysis is a systems biology approach providing correlation between all expressed genes across the samples and is performed by the R package WGCNA (Langfelder and Horvath 2008). Normalized data were used to construct co‐expression networks through WGCNA (Langfelder and Horvath 2008; Zhang and Horvath 2005). Gene expression values were normalized to transcripts per million (TPM) before network construction by RSEM. Two independent WGCNA were performed, one for skeletal muscle and another for liver. The resulting networks were subsequently integrated with the quantification of six cytokines, which were measured in both tissues. For the analysis of co‐expression networks, a Pearson correlation matrix was constructed, where the sign of correlation was preserved (“signed network”), which was converted to an adjacent matrix with adjusted correlation.

The Topological Overlap Matrix (TOM) was constructed based on dissimilarity measures (1–TOM) among nodes (genes) in conjunction with the hierarchical clustering method, considering a minimum number of 30 genes per module (Langfelder and Horvath 2007). In this step, genes were clustered by colors, and the grey module was clustered as non‐attributed genes to any module of co‐expression (Langfelder and Horvath 2008). Highly correlated modules were clustered based on the dissimilarity of their eigengenes, defined as the first principal component for each module, and considered a representation of the gene expression profile within the modules (Zhang and Horvath 2005).

Gene modules were characterized by dissimilarities lower than 0.25, corresponding to a correlation of 0.75. The co‐expression analysis was conducted on the module eigengene (ME) of each module and cytokine levels (IFN‐γ, IL‐1β, IL‐18, IL‐10, IL‐6, and TNF‐α).

Co‐expression networks were constructed separately for the skeletal muscle and liver, obtaining two different soft threshold powers. The soft threshold power is a parameter of WGCNA that transforms the gene expression correlations into a similarity matrix, used to build the co‐expression network. For both tissues, modules presenting Pearson's correlation ≥ |0.7| (nominal *p*‐value < 0.05) with at least one of the cytokines were considered in the further analysis. For functional enrichment analysis, the top 10 hub genes within relevant modules were selected. Nonetheless, some modules did not show enrichment; therefore, they were not included in the results and discussion sections.

### 2.6 Enrichment Analysis and Hub Genes

The top 10 hub genes were identified using the *cytoHubba* plugin (version 0.1) (Chin et al. 2014), using Maximal Clique Centrality (MCC). The results of the top 10 hub genes are detailed in Tables [Link], [Link], [Link], [Link] for muscle and liver. Based on these networks, protein–protein interaction (PPI) networks for the identified relevant modules (*Blue, Darkorange, Darkgreen*, and *Lightyellow*) were constructed using the STRING plugin (version 2.2.0) within Cytoscape (version 3.10.0) (Doncheva et al. 2018). Subsequently, Gene Ontology (GO) and pathway enrichment analyses were performed using the ClueGO plugin (version 2.5.10) (Bindea et al. 2009) to functionally characterize the hub genes. Significant GO terms and pathways were selected based on a Benjamini–Hochberg False Discovery Rate (FDR) < 0.05 (Benjamini and Hochberg 1995). In the Results section, we present the hub genes for which the most significant GO terms were identified.

## 3 Results

The total average number of sequenced reads for samples from the skeletal muscle for pigs fed SOY1.5 were 33 280 806 (28–38 M) and 16 398 815, before and after filtering, respectively. For pigs fed SOY3.0, the corresponding numbers were 33 215 576 (27–38 M) and 16 369 097. The total average number of reads from the liver of SOY1.5 pigs were 33 793 712 (28–46 M) and 16 656 851, and of the SOY3.0 pigs were 34 054 851 (29–41 M) and 16 788 537, before and after filtering, respectively.

### 3.1 WGCNA Was Performed in the Skeletal Muscle

**FIGURE 1:**
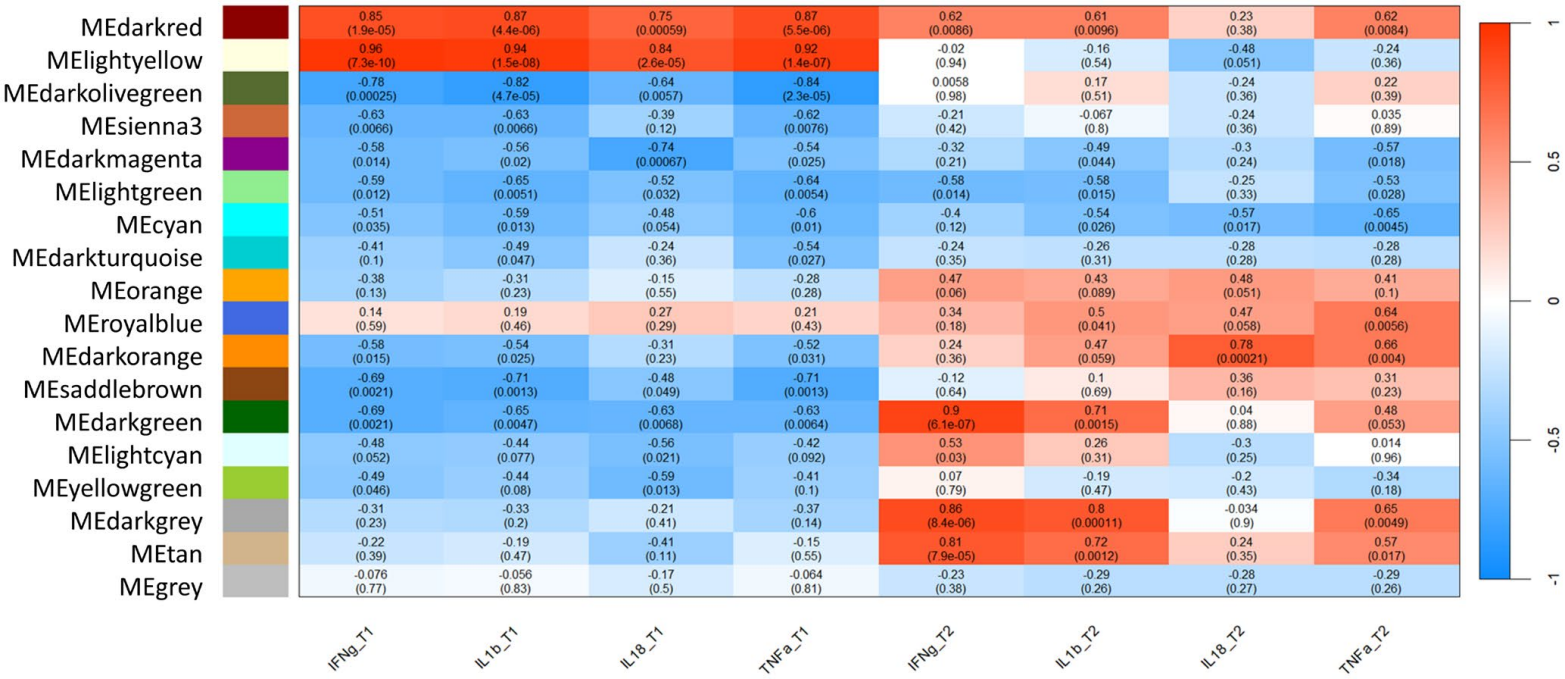
Heatmap obtained from WGCNA from skeletal muscle in pigs supplemented 1.5% (T1) and 3.0% (T2) soybean oil. The color gradient indicates the strength and direction of the correlation (red: Positive; blue: Negative), with corresponding *p*‐values shown in parentheses. Strong and significant correlations suggest potential functional associations between specific gene modules and cytokine responses. The Y‐axis represents module eigengenes, corresponding to clustered genes that were grouped into modules and distinguished by color. The X‐axis presents the cytokine levels for each diet. IFNg, Interferon‐γ; IL1b, Interleukin‐1β; IL, Interleukin; TNFa, Tumor necrosis factor‐α.

In the skeletal muscle, no statistical difference for the levels of cytokines in pigs fed SOY1.5 or SOY3.0 was found (Table S1). Growth performance and fatty acids profile presented statistical differences in average daily feed intake, eicosapentaenoic, and docosahexaenoic acids in the muscle, as previously demonstrated in Almeida, Silva, Meira, et al. (2021). WGCNA was performed using a soft threshold power equal to 20, following the soft threshold free topology scale. Relevant modules were defined by retaining gene clusters with a minimal Pearson correlation coefficient of |*r*| ≥ 0.7 (*p*‐value < 0.05). The WGCNA found 17 co‐expressed gene modules, which were associated with cytokine levels (Figure 1). IL‐10 and IL‐6 were removed from the dataset to prevent statistical noise, as these cytokines presented undetectable concentration levels across the majority of the skeletal muscle samples. We observed the highest positive correlation (*r* = 0.96 and *p*‐value = 7.3E−10) between the genes clustered in the *Lightyellow* module and IFN‐γ for pigs fed SOY1.5 (Figure 1).

Positively correlated modules with cytokines indicate both increased gene expression and cytokine levels (Langfelder and Horvath 2007). For the *Darkgreen* module, we observed the highest positive correlation (*r* = 0.9 and *p*‐value = 6.1E−07) with IFN‐γ in pigs fed SOY3.0. The presence of strong positive correlations between gene module and a trait demonstrates a co‐expression pattern between the clustered genes and IFN‐γ cytokine. This indicates that these genes are concomitantly upregulated.

The hub genes within the *Lightyellow* module showed strong associations with elevated expression of the four cytokines (IFN‐γ, IL‐1β, IL‐18, and TNF‐α), particularly in pigs fed SOY1.5. In contrast, IFN‐γ from pigs fed SOY3.0 was the only cytokine co‐expressed with the *Darkgreen* module (*r* = 0.9, *p*‐value = 6.1E−07).

### 3.2 Hub Genes in the Skeletal Muscle

**FIGURE 2:**
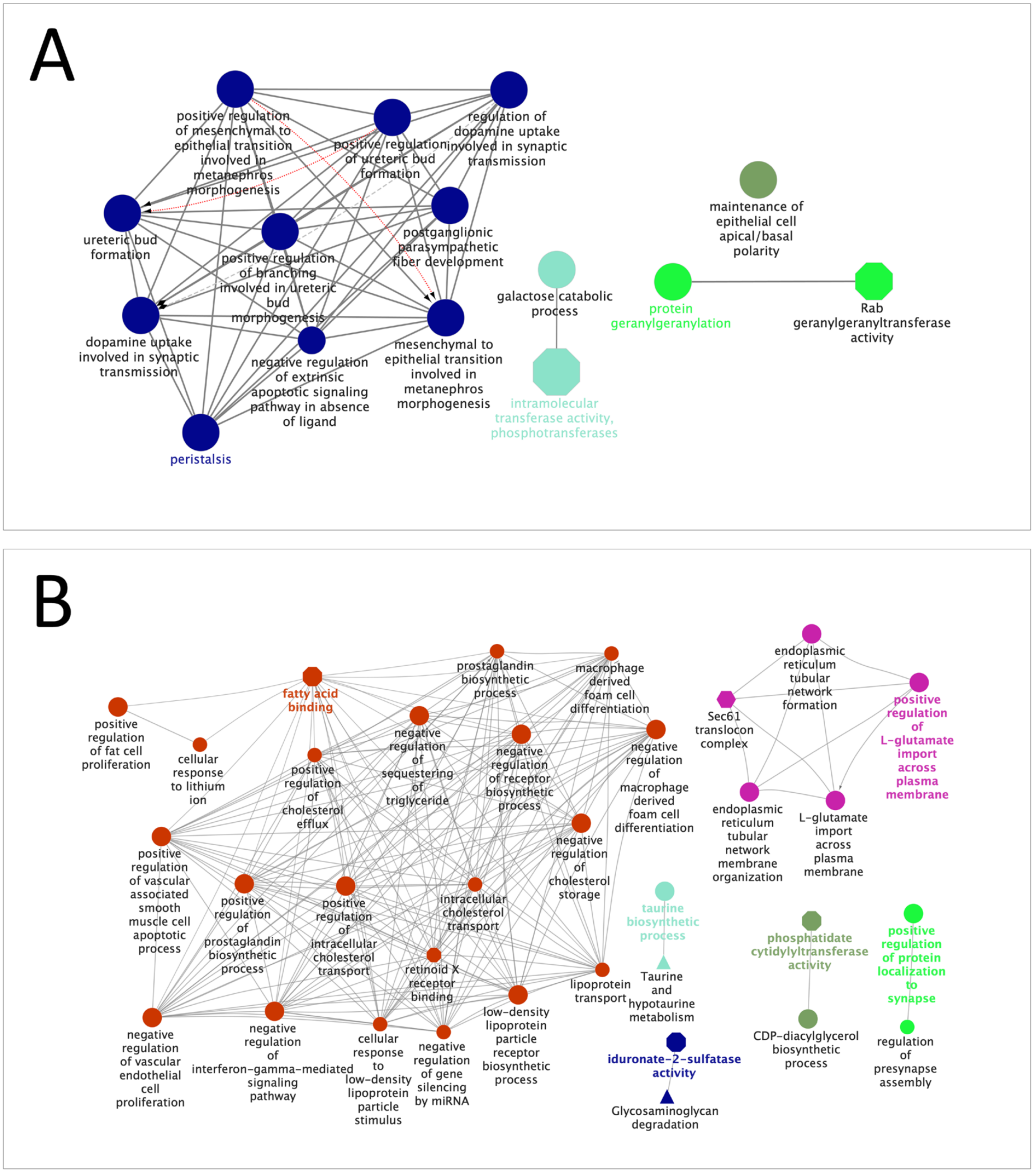
(A) Functional enrichment for skeletal muscle in the *Lightyellow* module correlated with IFN‐gamma in pigs supplemented with 1.5% soybean oil. (B) Functional enrichment for skeletal muscle in the *Darkgreen* module correlated with IFN‐gamma in pigs supplemented with 3.0% soybean oil. Symbols of triangle: KEGG Pathway; Circle: Biological Process GO term; Octagon: Molecular Function GO term; Hexagon: Cellular Component GO term.

The GO terms with FDR < 5% are shown in Table S2 for each module, for both the liver and in skeletal muscle. In the skeletal muscle, the functional enrichment of hub genes for the *Lightyellow* module (Figure 2A) revealed 15 GO terms from the top 10 hub genes (Table S3). The GO term “intramolecular transferase activity, phosphotransferases” showed the lowest FDR for this module. For the *Darkgreen* module, which has 34 GO terms associated with its top 10 hub genes (Table S4), the biological process “fatty acid‐binding” showed the lowest FDR (Figure 2B).

**FIGURE 3:**
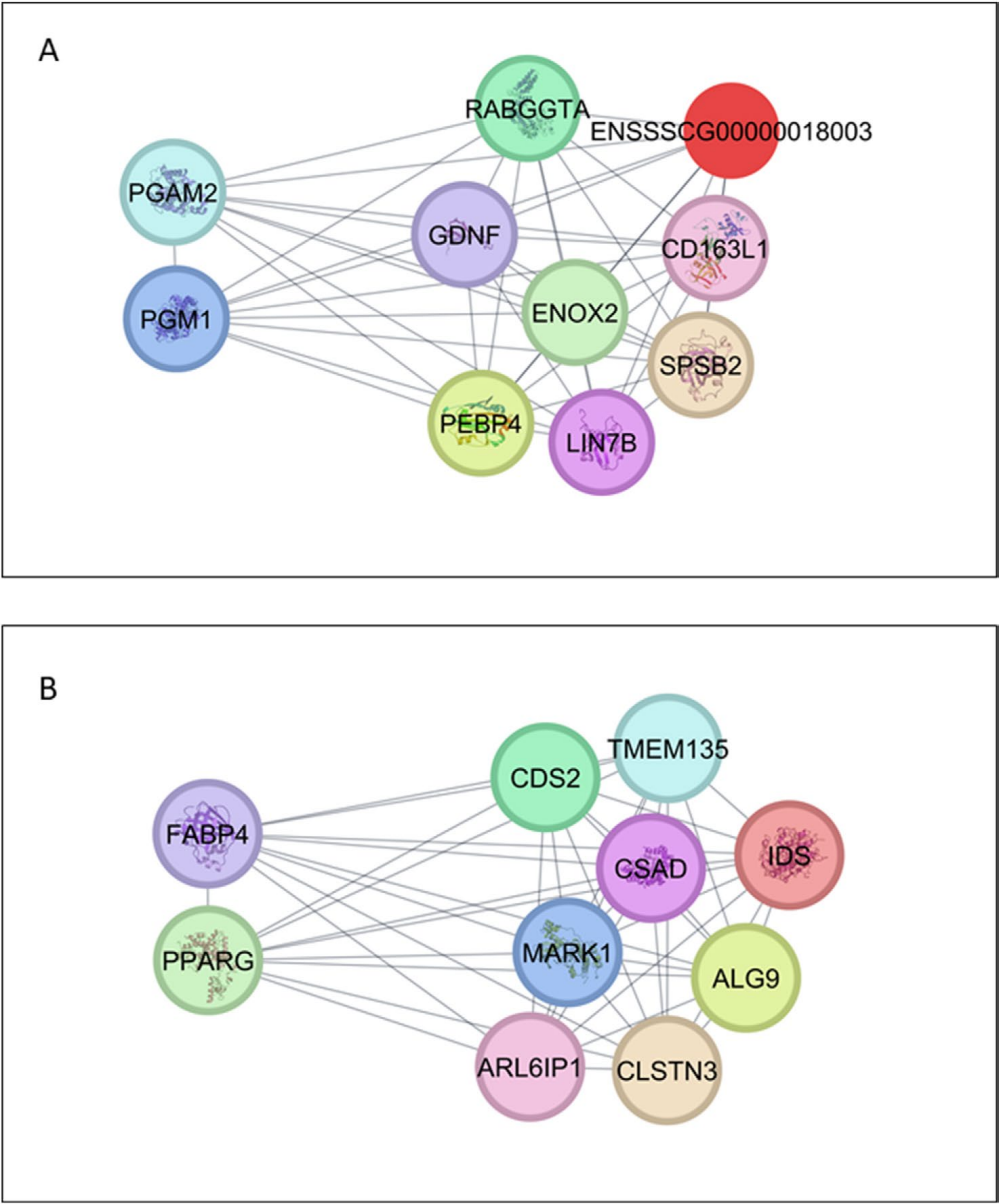
(A) The top 10 hub genes for the *Lightyellow* module highlighting *PGAM2* and *PGM1* as simultaneously the top 1. (B) The top 10 hub genes for the *Darkgreen* module highlighting *FABP4* and *PPARG* as simultaneously the top 1. Each circle represents a swine gene in the skeletal muscle; non‐annotated genes are presented as Gene Stable ID.

There were two hub genes identified in the “intramolecular transferase activity, phosphotransferases” GO term; they were Phosphoglucomutase 1 (*PGM1*) and Phosphoglycerate Mutase 2 (*PGAM2*) (Figure 3A), both of which regulate intramolecular transferase activity in skeletal muscle. All cytokines analyzed in this module presented high correlations: IFN‐γ (*r* = 0.96), IL‐1β (*r* = 0.94), IL‐18 (*r* = 0.84), and TNF‐α (*r* = 0.92). These four cytokines are pro‐inflammatory and are positively co‐expressed with *PGM1* and *PGAM2* in pigs only fed SOY1.5.

On the *Darkgreen* module, there were two hub genes identified in the “fatty acid‐binding” GO term; they were the Fatty Acid‐Binding Protein 4 (*FABP4*) and the Peroxisome Proliferator‐Activated Receptor Gamma (*PPARG*) (Figure 3B). The cytokine strongly associated with these *FABP4* and *PPARG* genes was IFN‐γ for pigs only fed SOY3.0.

### 3.3 WGCNA Was Performed in the Liver Tissue

**FIGURE 4:**
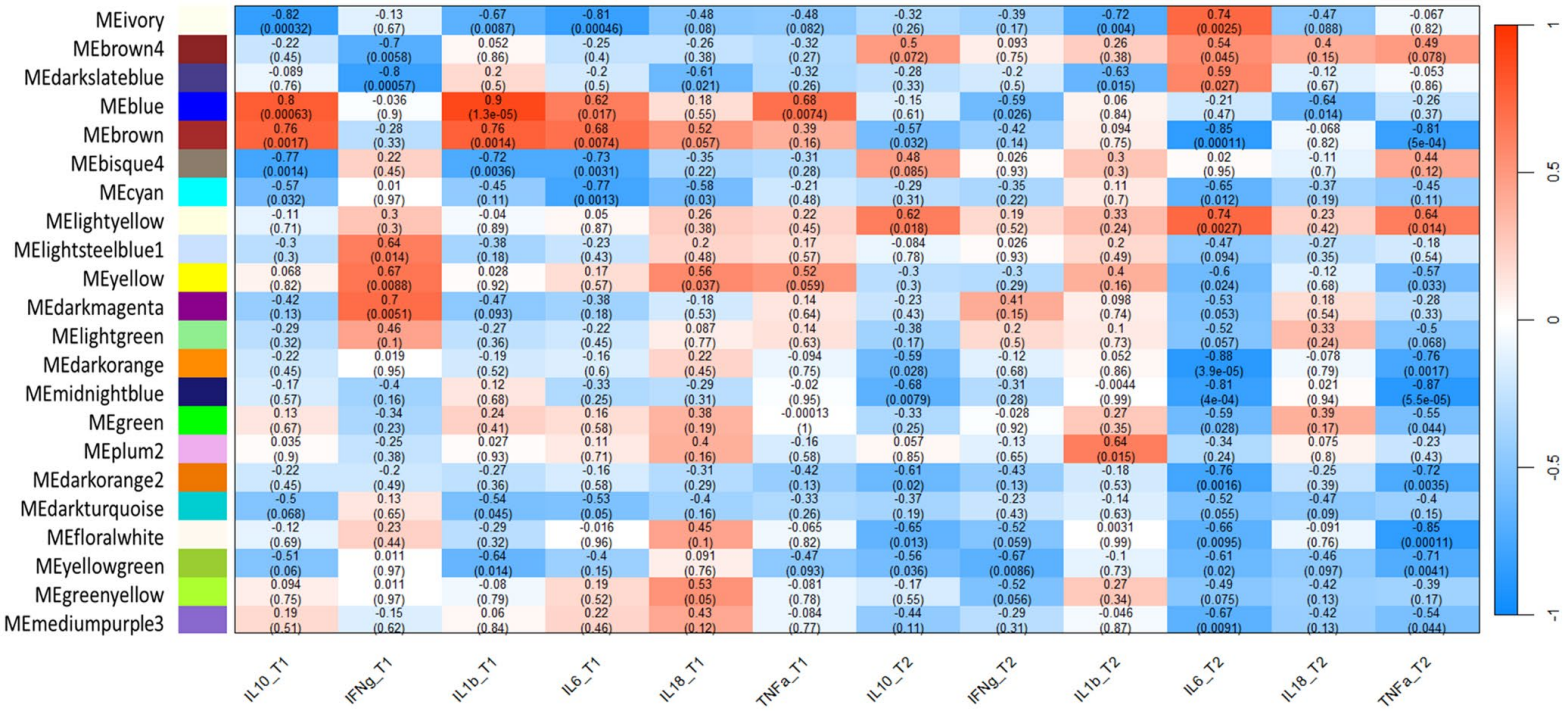
Heatmap obtained from WGCNA from liver in pigs supplemented 1.5% (T1) and 3.0% (T2) soybean oil. The color gradient indicates the strength and direction of the correlation (red: Positive; blue: Negative), with corresponding *p*‐values shown in parentheses. Strong and significant correlations suggest potential functional associations between specific gene modules and cytokine responses. The Y‐axis represents module eigengenes, corresponding to clustered genes that were grouped into modules and distinguished by color. The X‐axis presents the cytokine levels for each diet. IFNg, Interferon‐γ; IL1b, Interleukin‐1β; IL, Interleukin; TNFa, Tumor necrosis factor‐α.

No statistical difference among cytokines was found in the liver for the pigs fed SOY1.5 and SOY3.0 (Table S5). WGCNA analysis was also conducted for the liver, with the liver network constructed using a soft threshold power of 12 following the soft threshold free topology scale. WGCNA assigned 22 modules as associated with the cytokines of interest (Figure 4).

In the liver of pigs fed SOY1.5, WGCNA identified two positive correlations for the *Blue* module: IL‐10 (*r* = 0.80, *p*‐value = 0.00063) and IL‐1β (*r* = 0.90, *p*‐value = 1.3E−05). In pigs fed SOY3.0, a negative correlation was observed for the *Darkorange* module and IL‐6 (*r* = −0.88, *p*‐value = 3.9E−05) (Figure 4). Although other correlations exceeded *r* > 0.7, such as in the *Darkorange* module with TNF‐α for SOY3.0 (*r* = −0.76 and *p*‐value = 0.0017), we focused on the correlation with IL‐6, given that this was the highest correlation observed.

### 3.4 Hub Genes in the Liver

**FIGURE 5:**
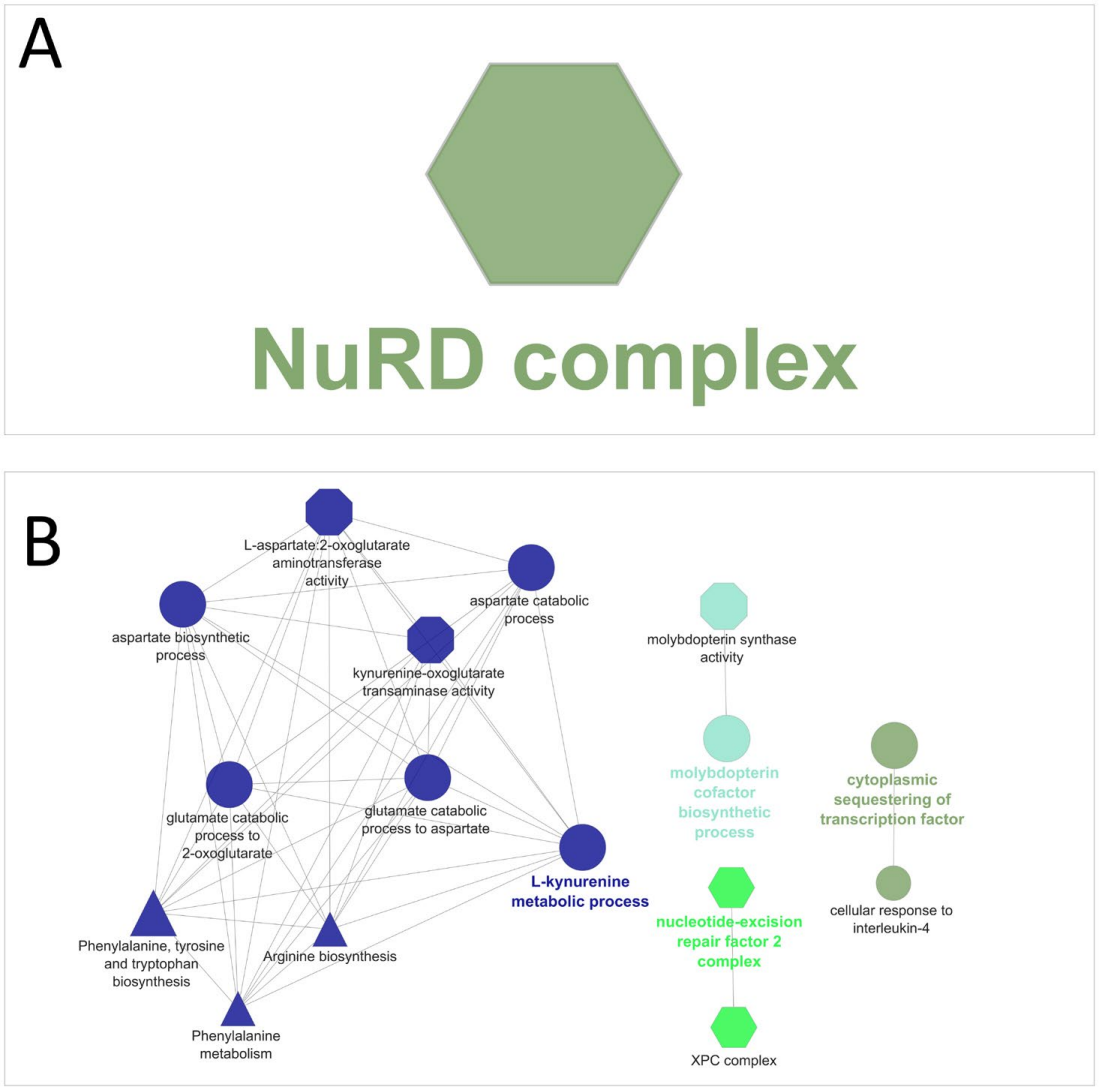
(A) Functional enrichment of the Cellular Component GO term for liver tissue in the *Blue* module correlated with IL‐1β in pigs supplemented with 1.5% soybean oil. (B) Functional enrichment for liver in the *Darkorange* module correlated with IL‐6 in pigs supplemented with 3.0% soybean oil. Symbols of triangle: KEGG Pathway; Circle: Biological Process GO term; Octagon: Molecular Function GO term; Hexagon: Cellular Component GO term.

The GO terms with FDR < 5% are shown in Table S2 for each module shown in this section for liver and the diets SOY1.5 and SOY 3.0. Regarding functional enrichment of the *Blue* module in the liver, the top 10 hub genes (Table S6) were enriched for one cellular component term, the “NuRD complex” (Figure 5A). The top 10 hub genes (Table S7) in *Darkorange* were enriched for “cellular response to IL‐4” (Figure 5B).

**FIGURE 6:**
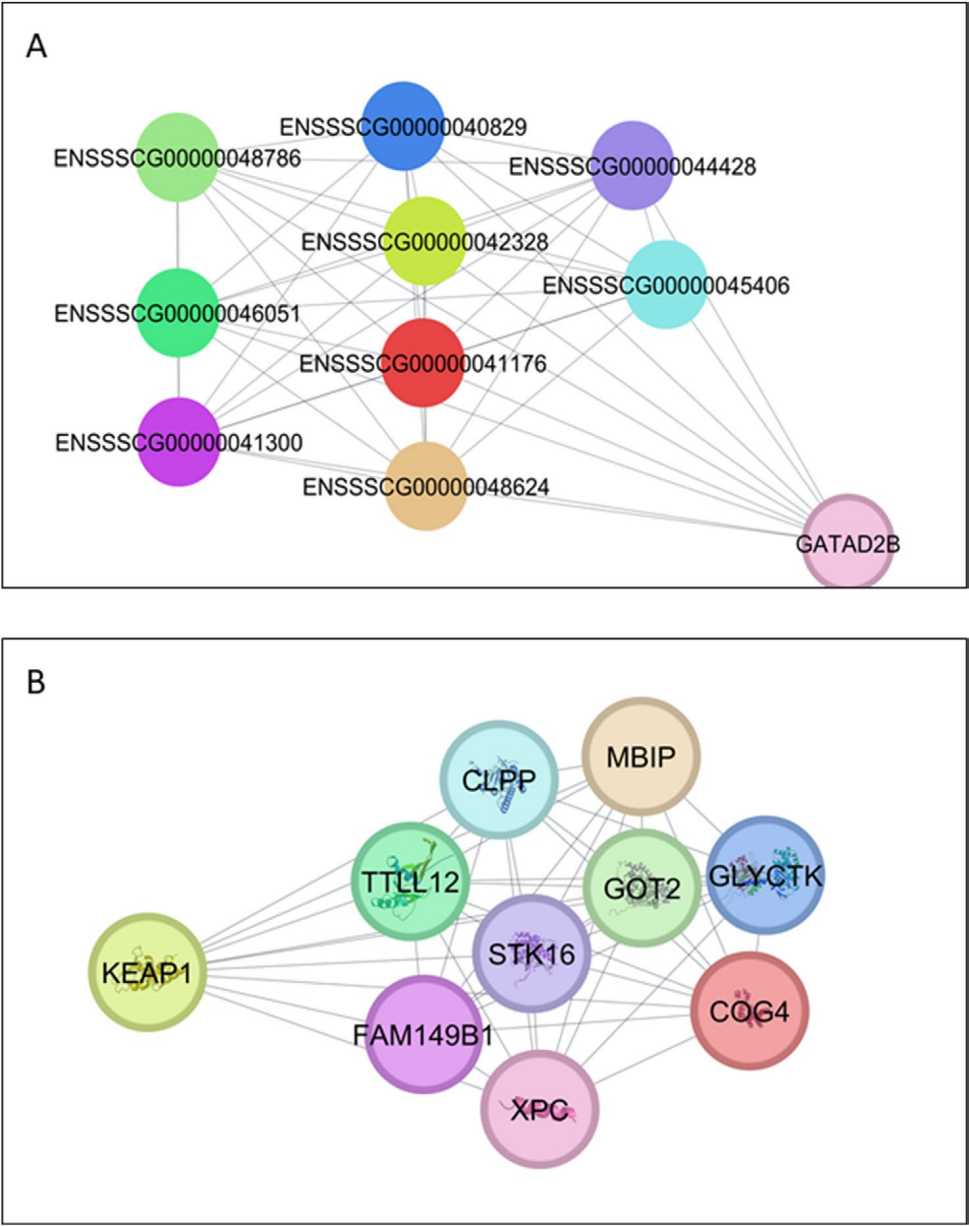
(A) The top 10 hub genes for the *Blue* module highlighting the *GATAD2B* as the top 1. (B) The top 10 hub genes for the *Darkorange* module highlighting the *KEAP1* as the top 1. Each circle represents a swine gene in the liver; non‐annotated genes are presented as Gene Stable ID.

The hub gene, GATA Zinc Finger Domain Containing 2B (*GATAD2B*) (Figure 6A), was enriched for the ClueGO plug‐in in the *Blue* module, and this gene is related to “NuRD complex”. Another hub gene, Kelch Like ECH Associated Protein 1 (*KEAP1*) (Figure 6B), was enriched for the biological process GO term “cellular response to interleukin‐4.” This hub gene, *KEAP1*, was strongly negatively associated with IL‐6 (*r* = −0.88).

## 4 Discussion

This study demonstrated that both tissues exhibit dynamic biological processes modulated by soybean oil concentration. In summary, the lower concentration (SOY1.5) intensified gene regulation and glucose metabolism in skeletal muscle, while also favoring epigenetic mechanisms in the liver. In contrast, the higher concentration (SOY3.0) promoted benefits related to hepatic oxidative stress and the regulation of lipid‐associated genes in skeletal muscle. Therefore, oxidative stress pathways, fatty acids, gene methylation, and glucose metabolism emerge as central to understanding cytokine profiles in healthy pigs, underscoring the relevance of lipid nutrition in modulating immunological and metabolic processes that directly impact animal performance and health.

Skeletal muscle tissue exhibited two highly significant correlations with the *Darkgreen* and *Lightyellow* modules, which were related to GO terms “fatty acid‐binding” and “intramolecular transferase activity, phosphotransferases,” respectively. In the *Darkgreen* module, a positive correlation was observed with IFN‐γ (*r* = 0.9) in pigs fed SOY3.0. IFN‐γ is a pro‐inflammatory cytokine regulated by the transcription factor NFkB, capable of inducing immunomodulatory responses at the gene expression level by controlling genes that contain STAT1 binding sites, such as those related to antigen presentation (Alspach et al. 2018; Teixeira et al. 2005). Moreover, IFN‐γ can be modulated by inhibitors of fatty acid synthesis in certain immunological disorders (Iwata et al. 2020).

Although fatty acids are associated with metabolic diseases such as diabetes, monounsaturated fatty acids (MUFAs) can prevent inflammation and insulin resistance in skeletal muscle by suppressing the AMPK pathway (Radzikowska et al. 2019). The proportion of essential fatty acids in castrated pigs or young gilts may directly influence growth performance (Becker et al. 2023). In this context, omega (n)‐3 fatty acids contribute to cell membrane fluidity and respond to thermal, dietary, or inflammatory stimuli by regulating cytokine secretion or production (Ali and Szabó 2023; Gutiérrez et al. 2019).

Within the GO term “fatty acid‐binding,” two hub genes were identified: *FABP4* and *PPARG*. Fatty acid‐binding proteins (FABPs) participate in systemic or tissue‐specific inflammation and are associated with high‐fat diets, obesity, diabetes, dyslipidemia, and atherosclerosis. *FABP4*, a cytoplasmic protein capable of binding long‐chain fatty acids (LCFAs), was positively regulated by the increase of these fatty acids present in soybean oil. In addition to playing a role in fatty acid uptake during obesity or fatty liver disease, these proteins can also activate transcriptional factors such as *PPARG* (Furuhashi and Hotamisligil 2008; Li et al. 2022; Mallick et al. 2023). *PPARG*, in turn, functions as an anti‐inflammatory mediator and glucose regulator (Welch et al. 2003), and can dimerize with RXR to modulate the expression of lipogenic genes (Fanalli, da Silva, Gomes, Ciconello, et al. 2022).

The interaction between IFN‐γ and *PPARG* reveals an important balance: IFN‐γ can reduce lipoprotein lipase activity and *PPARG* expression, thereby increasing lipolysis in adipocytes (Waite et al. 2001). Some enzymes, such as lipoxygenases and prostaglandin synthases, can activate *PPARG*, thus downregulating IFN‐γ‐dependent genes (Welch et al. 2003). Therefore, a cross‐regulatory network between fatty acids, cytokines, and transcriptional factors can be observed in skeletal muscle.

In the *Lightyellow* module, with the “intramolecular transferase activity, phosphotransferases” as GO term presenting lower FDR, all cytokines analyzed exhibited a strong positive correlation with skeletal muscle under SOY1.5 treatment. The highest correlation was observed with IFN‐γ (*r* = 0.98), followed by IL‐1β (*r* = 0.94), TNF‐α (*r* = 0.92), and IL‐18 (*r* = 0.84). In the “intramolecular transferase activity, phosphotransferases” GO term, two hub genes were identified: *PGM1* and *PGAM2* (Table S2). Phosphotransferases, in turn, can couple transmembrane transport with concomitant phosphorylation of carbohydrates (Roth et al. 2024). Since phosphoproteins are involved in the transduction of extracellular signals in muscle cells (Toutant and Sobel 1987), glycolysis assumes a central role, as it can enhance the production of pro‐inflammatory cytokines such as TNF‐α, IL‐1β, and IFN‐γ (Soto‐Heredero et al. 2020). This process may be particularly relevant for finishing pigs, which require high energy metabolism to sustain growth.

The expression of these pro‐inflammatory cytokines can be modulated by glycolytic molecules and by high levels of intracellular acetyl‐CoA, which enter the nucleus and enhance gene expression through DNA acetylation (Soto‐Heredero et al. 2020). Moreover, IFN‐γ can regulate amino acid phosphorylation in the brain and affect tryptophan catabolism, thereby increasing fatty acid oxidation in the endothelium or activating gene expression pathways (Lee et al. 2021; Li et al. 2015; Nair et al. 2002). Thus, it is suggested that the lower concentration of soybean oil strongly modulates glucose metabolism and the activation of pro‐inflammatory cytokines in skeletal muscle.

Furthermore, in hepatic tissue, four significant correlations were observed in the *Blue* and *Darkorange* modules, which were associated with “cellular response to IL‐4” and “NuRD complex” GO terms, respectively. In the *Blue* module, IL‐10 and IL‐1β showed strong positive correlations (*r* = 0.8 and *r* = 0.9, respectively) with the SOY1.5 diet. These cytokines can be associated with epigenetic regulation and pathological processes in the liver (Li et al. 2020; Zhang and Kuchroo 2019). IL‐1β, a pro‐inflammatory cytokine, is linked to tumor aggressiveness, heart failure, and local inflammation, frequently associated with altered methylation patterns in promoter regions (Butts et al. 2018; Hashimoto et al. 2009). IL‐10, in contrast, is anti‐inflammatory and regulates B‐ and T‐cell responses as well as epigenetic mechanisms (Huang et al. 2016). The hub gene *GATAD2B*, which is strongly associated with both IL‐1β and IL‐10 cytokines, may play a crucial role in epigenetic regulation, functioning as a transcriptional repressor within the “NuRD complex” and potentially implicated in metabolic diseases and cancer (Reid et al. 2023; Jiang et al. 2024; Liu et al. 2019).

While the SOY1.5 diet may be modulating epigenetic processes, results for the SOY3.0 diet suggest that it provides antioxidant protection, indicating distinct effects in the liver. Based on the *Darkorange* module, the most significant GO term was “cellular response to IL‐4,” which is related to methylation across different cell types (Kastner et al. 2010; Vento‐Tormo et al. 2016). In this module, IL‐6 and TNF‐α were negatively correlated in the liver under SOY3.0 supplementation (*r* = −0.88 and *r* = −0.76, respectively). The hub gene *KEAP1*, a regulator of antioxidant responses, is connected to the Nrf2 pathway and recognized as a defense mechanism against oxidative stress (Matsuoka et al. 2016).

Both SOY1.5 and SOY3.0 treatments produced important outcomes in a genetic and metabolic context, indicating that the interaction between treatments may influence important physiological responses between cytokines and gene expression across the two tissues in healthy pigs fed different concentrations of soybean oil. These findings highlight the need for more comprehensive studies integrating epigenetics, oxidative stress, glucose metabolism, and lipidomics to deepen our comprehension about mechanisms underlying inflammation and lipid metabolism in pigs.

## Author Contributions

**Fernanda Nery Ciconello:** conceptualization, formal analysis, funding acquisition, investigation, methodology, writing – original draft, writing – review and editing. **Julia Dezen Gomes:** writing – review and editing. **Simara Larissa Fanalli:** writing – review and editing. **Bárbara Silva‐Vignato:** writing – review and editing. **Andrezza Maria Felício‐Ament:** writing – review and editing. **Vivian Vezzoni de Almeida:** writing – review and editing. **Cristina Tchorny Moncau‐Gadbem:** writing – review and editing. **James Eugene Koltes:** writing – review and editing. **Christopher Keith Tuggle:** conceptualization, supervision, writing – review and editing. **Aline Silva Mello Cesar:** conceptualization, formal analysis, funding acquisition, investigation, methodology, project administration, resources, supervision, writing – original draft, writing – review and editing.

## Funding

We acknowledge FAPESP, CAPES, and CNPq for providing all funding support. This study was supported by the São Paulo State Research Foundation (FAPESP, grant numbers: A.S.M.C. 2017/25180‐2, F.N.C. 2021/10394‐2, F.N.C. 2022/13606‐3). This study was financed in part by the Coordenação de Aperfeiçoamento de Pessoal de Nível Superior—Brazil (CAPES)—Finance Code 001. The Brazilian National Council for Scientific and Technological Development (CNPq, grant number: A.S.M.C. 303165/2022‐7).

## Ethics Statement

The experiment and sample collection were approved according to the established requirements by the Council for Experiments on Animals, and it was approved by the Committee of Animal Use and Ethics from “Luiz de Queiroz” College of Agriculture at the University of Sao Paulo (CEUA/ESALQ/USP). The registered protocol was 2018.5.1787.11.6 and CEUA number 2018‐28.

## Conflicts of Interest

The authors declare no conflicts of interest.

## Supporting information


**Table S1:** Statistical test for skeletal muscle comparing SOY1.5 and SOY3.0.

**Table S2:** GO terms of the four modules in the skeletal muscle and liver with an FDR < 5% in pigs fed SOY1.5 and SOY3.0.

**Table S3:** Top 10 in network CytoscapeInput‐edges‐darkgreen ranked by MCC method in the skeletal muscle for SOY3.0.

**Table S4:** Top 10 in network CytoscapeInput‐edges‐lightyellow ranked by MCC method in the skeletal muscle for SOY1.5.

**Table S5:** Statistical test for liver comparing SOY1.5 and SOY3.0.

**Table S6:** Top 10 in network CytoscapeInput‐edges‐blue ranked by MCC method in the liver for SOY1.5.

**Table S7:** Top 10 in network CytoscapeInput‐edges‐darkorange ranked by MCC method in the liver for SOY3.0.

## Data Availability

The dataset used in this study is available in the European Nucleotide Archive (ENA) repository (EMBL‐EBI), under accession PRJEB50513 https://www.ebi.ac.uk/ena/browser/view/PRJEB50513, and accession PRJEB52629. https://www.ebi.ac.uk/ena%20/browser/view/PRJEB52629.
